# Weekly Observations of Estuarine Microbial Assemblages during Summer in the Inner Part of Ariake Bay, Japan; Microbial Water-sediment Coupling in Turbid Shallow Waters

**DOI:** 10.1264/jsme2.ME22015

**Published:** 2022-06-08

**Authors:** Ryo Orita, Kazuhiro Yoshida, Hiroto Terazono, Yukio Nagano, Masatoshi Goto, Kei Kimura, Genta Kobayashi

**Affiliations:** 1 Faculty of Agriculture, Saga University, 1 Honjo-machi, Saga, 840–8502, Japan; 2 Analytical Research Center for Experimental Sciences, Saga University, 1 Honjo-machi, Saga, 840–8502, Japan

**Keywords:** environmental factors, microbial diversity, high-throughput sequencing, benthic microbes, pelagic microbes

## Abstract

Estuarine microbial assemblages are altered by a number of environmental factors, and knowledge of these changes is essential for understanding the functions of microbes in estuarine ecosystems. The aims of the present study were to examine the relationship between microbial assemblages in the water column and sediment surface, and to identify the environmental factors that influence the short-term dynamics of microbial assemblages in these two zones in summer in the inner part of Ariake Bay. The microbial assemblage of each sample consisted of a mean of 71.1% operational taxonomic units (OTUs), which commonly occurred in the water column and sediment surface, although their relative composition markedly differed between the two zones. In the water column, spatiotemporal changes in microbial assemblages correlated with several environmental factors, such as the nitrogen content in suspended particles, turbidity, and salinity. On the other hand, temporal changes in the sediment’s microbial assemblages were governed by a single environmental factor, namely, the oxygen reduction potential. These results suggest that the composition of microbial assemblages in the water column and sediment surface differed even in highly turbid brackish waters with high sediment resuspension, and the environmental factors contributing to the change in the assemblage composition also differed between the water column and sediment.

Ocean biogeochemical cycles are primarily governed by interactions between autotrophic and heterotrophic microbes (Sarmiento and Gruber, 2006); autotrophic phytoplankton actively produces organic matter within the filmly sun-lit layer, whereas heterotrophic bacteria, which are distributed from the surface water to the sediment, decompose organic matter by using it as a respiratory substrate. Due to the supply of diverse organic substrates, microbial processes are complex; each microbial species that specialize in different material cycles coexist in water and sediment (*e.g.*, remineralization, nitrification, and sulfur reduction) (Kaku *et al.*, 2005; Baker *et al.*, 2012; Kirchman, 2012; Sakami, 2012). Consequently, the diversity and abundance of marine microbes are huge (>a million species and ~300 Pg of biomass) (Whitman *et al.*, 1998; Curtis *et al.*, 2002). Ocean physics and chemistry modify the distribution and activity of microbial communities, both in the water column and in sediment (Herlemann *et al.*, 2011; Mahmoudi *et al.*, 2015; Khandeparker *et al.*, 2017; Qian *et al.*, 2018). Dynamic coastal waters are one of the most suitable settings to study the interactions between microbes and the environment because they are characterized by sporadic large variations in temperature and salinity as well as by active organic matter production. In addition to sporadic large variations, water-sediment coupling stimulated by tidal mixing may be a significant factor controlling coastal microbial communities. The significant resuspension of sediment may modify physical and chemical properties, as well as the vertical microbial distribution, consequently affecting biogeochemical cycles and biological activities.

Ariake Bay is a semi-enclosed bay located on the western coast of Kyushu in western Japan, and has the largest tidal range in the country, which reaches a maximum of more than 6‍ ‍m in the innermost area. There is an evident salinity gradient from the inner part of the bay to the bay mouth (Tsutsumi, 2006; Yanagi and Shimomura, 2006). Furthermore, a strong halocline is known to develop in the inner part of the bay, particularly during the rainy season between June and July (Tsutsumi, 2006; Orita *et al.*, 2015). Moreover, environmental factors, such as water temperature, chlo­rophyll *a* (Chl-*a*), and dissolved oxygen (DO), differ between the upper and lower layers of the halocline in summer (Orita *et al.*, 2015). The strong tidal current significantly resuspends sediment particles in the inner part of the bay at which mud flats are located, forming high-turbidity waters (Sato, 2010). Due to the large variations in turbidity associated with the cycle of spring and neap tides, the amount of sediment resuspended in the water column also changes in the short term, namely, every other week (Koh *et al.*, 2006; Tanaka and Kodama, 2007; Hayami *et al.*, 2019). As a result, environmental fluctuations frequently (*i.e.*, weekly to monthly) occur in Ariake Bay, which modify microbial assemblages in the water column and sediment surface, particularly in the inner part of Ariake Bay in summer.

In the present study, weekly field surveys were conducted on four water layers (*i.e.*, two layers above and below the halocline) and also on the sediment surface. The aims of this study were as follows: 1) to examine the relationship between microbial assemblages in the water column and sediment surface, and 2) to identify environmental factors that influence the short-term dynamics of microbial assemblages in both zones. In addition, the composition of their operational taxonomic units (OTUs) was compared every other week to identify relationships between the water column and sediment microbial assemblages in turbid estuary waters. Furthermore, the ana­lysis considered the environmental factors contributing to spatiotemporal changes in microbial assemblages in the water column and on the sediment surface in summer in the inner part of Ariake Bay.

## Materials and Methods

### Field surveys

The study area is located in the inner part of Ariake Bay, Japan. To accurately capture the fluid marine environment, three replication sites were established within the study area (Fig. S1). The three sites, located approximately 400‍ ‍m from each other, were arranged in a triangle and data on environmental factors in each survey were representative of the average of data from these three sites. In the ana­lysis of microbial assemblages, the Site B sample was used as a representative of the study area (Fig. S1). The depth of the study area ranged between 12 and 15‍ ‍m depending on the tide. A total of four field surveys were conducted, once every week between July 13, 2018 and August 3, 2018. At each sampling site, the vertical profiles of temperature, salinity, DO, turbidity, and Chl-*a* were measured every meter along the depth profile using multiple water quality sensors (AAQ1183; JFE Advantech), and sediment and water samples were also collected. Specifically, the latter were collected using a Van Dorn water sampler (Rigo) on the surface, at a depth of 2 m, as well as 2 and 0.5‍ ‍m above the sea floor, and were stored separately for a microbial ana­lysis (660‍ ‍mL) and particulate organic matter (POM) quantification (100‍ ‍mL). The water samples to be used for the microbial assemblage ana­lysis were filtered through a 10-μm plankton net to remove large particles before storage in plastic bottles. Sediment samples were collected using a Smith-McIntyre grab sampler (22.5×22.5‍ ‍cm^2^), and 10 plastic core samplers (diameter: 29‍ ‍mm) were used to collect surface sediment up to a depth of 0.5‍ ‍cm, which were homogenized in a plastic bag to form sediment samples. The *in situ* oxygen redox potential (ORP) of surface sediment was measured with an ORP meter (RM30P, DKK-TOA). All water and sediment samples were kept in a cooler box and brought back to the laboratory.

### Sample ana­lysis

Water samples for the microbial ana­lysis were filtered through membrane filters (cellulose acetate membrane filters, Advantec, 47‍ ‍mm in diameter, pore size 0.2‍ ‍μm), and microbial DNA was extracted from the residues on the filters using an ISOIL for the Beads Beating kit (Nippon Gene). In the ana­lysis of microbial assemblages, the V4–V5 region of the 16S rRNA gene was sequenced with the primer pair 515F (5′-GTGCCAGCMGCCGCGG-3′) and 907R (5′-CCGTCAATTCCTTTGAGTTT-3′). Paired-end libraries (paired-end sequenced 250×2) were sequenced using an Illumina HiSeq 2500 platform. The amount of sequence data for all samples was more than 100,000 reads (Mean: 142,660 reads, Max: 159,741 reads, Min: 113,310 reads). Raw sequencing data were deposited in DDBJ as DRA013351.

Water samples for the POM quantification were filtered through pre-combusted glass-fiber filters (25‍ ‍mm in diameter; Whatman), which were then freeze-dried. After inorganic carbonates were eliminated with HCl, particulate organic carbon (POC) and particulate nitrogen (PN) were measured using a carbon-hydrogen/nitrogen (CHN) elemental analyzer (CHN coder JM-10; J-Science Lab).

Following the adequate homogenization of sediment samples, subsamples were divided to perform microbial (0.5 g) and physicochemical ana­lyses. DNA extraction and DNA sequence ana­lyses of sediment samples were performed using the same procedures as those for water samples. The physicochemical characteristics of sediment samples were evaluated in terms of the mud content, total organic carbon (TOC) and total nitrogen (TN) contents, and acid-volatile sulfide (AVS). The mud content of the sediment was assessed by wet sieving (the fraction <63‍ ‍μm). In the ana­lysis of TOC and TN contents, the sample was freeze-dried and powdered. After the removal of inorganic carbonates with HCl, the sample was re-dried, powdered, and TOC and TN contents were then measured using a CHN elemental analyzer. The measurement of AVS was conducted based on the methods described in Orita *et al.* (2015).

### Data ana­lysis

Reads were processed using the bioinformatic platform QIIME2, version 2019.7. Barcodes and adapter sequences and reads with low quality were removed using the “trimmomatic” tool (Bolger *et al.*, 2014). Trimmed sequence data (threshold for trimming: ≥99%) were converted into the fasta format for taxonomic annotation. Taxonomic classification was conducted through the “Greengene” database, version gg-13-8-99nb-classifier.qza, released in 2013, with >97% similarity based on the 16S rRNA V4–V5 region.

To evaluate the similarity of the microbial assemblage composition, a cluster ana­lysis based on the Bray–Curtis similarity (Bray and Curtis, 1957) was performed using the group average method. A redundancy ana­lysis (RDA) was conducted to examine the primary environmental factors (temperature, salinity, DO, turbidity, Chl-*a*, POM, PN, and CN ratio) affecting microbial assemblages in the water column during the sampling period. Regarding data related to these environmental factors, the average obtained from the three replication sites was used as the representative value for the area (Table S1). In the RDA ana­lysis, the Hellinger transformation was applied to microbial assemblage data (Legendre and Gallagher, 2001). The significance of the relationships between microbial assemblages and environmental factors was assessed by Monte Carlo permutation tests (999 randomized runs). PERMANOVA, with the Adonis function in the R package vegan, was used to examine the significance of differences between the microbial assemblage groups classified by RDA (Anderson, 2001). The SIMPER ana­lysis was performed to evaluate the OTUs that contributed to differences between microbial assemblage groups in the sediment. Differences between the environmental factors of the sediment surface were tested using the Wilcoxon rank-sum test. All multivariate and statistical ana­lyses were performed in R (version 4.0.3).

## Results

### Relationship between microbial assemblages in the water column and in the sediment

**Fig. 1.**
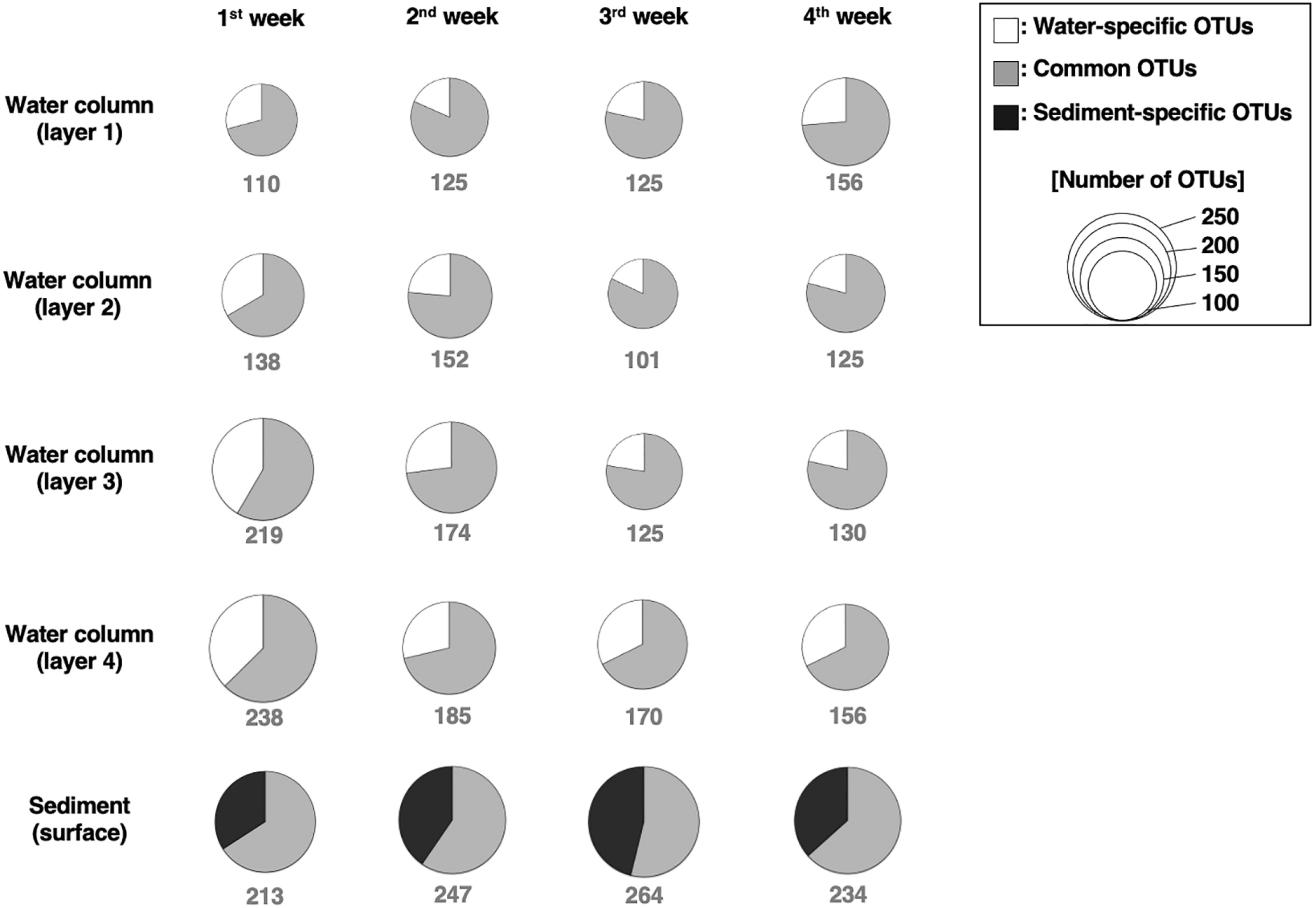
Spatiotemporal changes in the composition of OTUs during the monitoring period. The horizontal direction indicates the sampling date (1^st^ week: July 13, 2018; 2^nd^ week: July 20, 2018; 3^rd^ week: July 27, 2018; and 4^th^ week: August 3, 2018). The vertical direction indicates the sampling layer of the water column (layer 1: sea surface, layer 2: 2‍ ‍m in depth, layer 3: 2‍ ‍m above the sea floor, and layer 4: 0.5‍ ‍m above the sea floor) and seafloor (0.5‍ ‍cm in depth from sediment surface). The number below the pie chart indicates the total number of OTUs.

The number of OTUs included in the microbial assemblages in each sample ranged between 101 and 264, with a mean OTU count of 169.4 (Fig. 1). The total OTU count did not markedly change among sampling days or in the water column or surface sediment. The total OTU count varied with depth, with the maximum value in the water column being recorded in the 0.5-m layer above the seafloor every sampling day (layer 4 in Fig. 1). In addition, the total OTU count was slightly higher in surface sediment than in the water column.

The OTUs detected in both the water column and surface sediment accounted for 71.1% of all samples on average, 58.9 to 83.2% of the total OTUs in the water column, and 54.2 to 66.2% of the total OTUs in the sediment. The number of OTUs detected only in the water column ranged between 17 and 90 (16.8 to 41.1% of the total number of OTUs), and their proportion was slightly higher in the 0.5-m layer above the seafloor. The number of OTUs detected only in surface sediment ranged between 72 and 121, accounting for between 33.8 and 45.8% of the total number of OTUs occurring in sediment. OTUs detected only in the water column or surface sediment were markedly smaller as a relative composition of the microbial assemblage (water-specific OTUs: 1~6%, sediment-specific OTUs: 7~13%, Fig. S2).

**Fig. 2.**
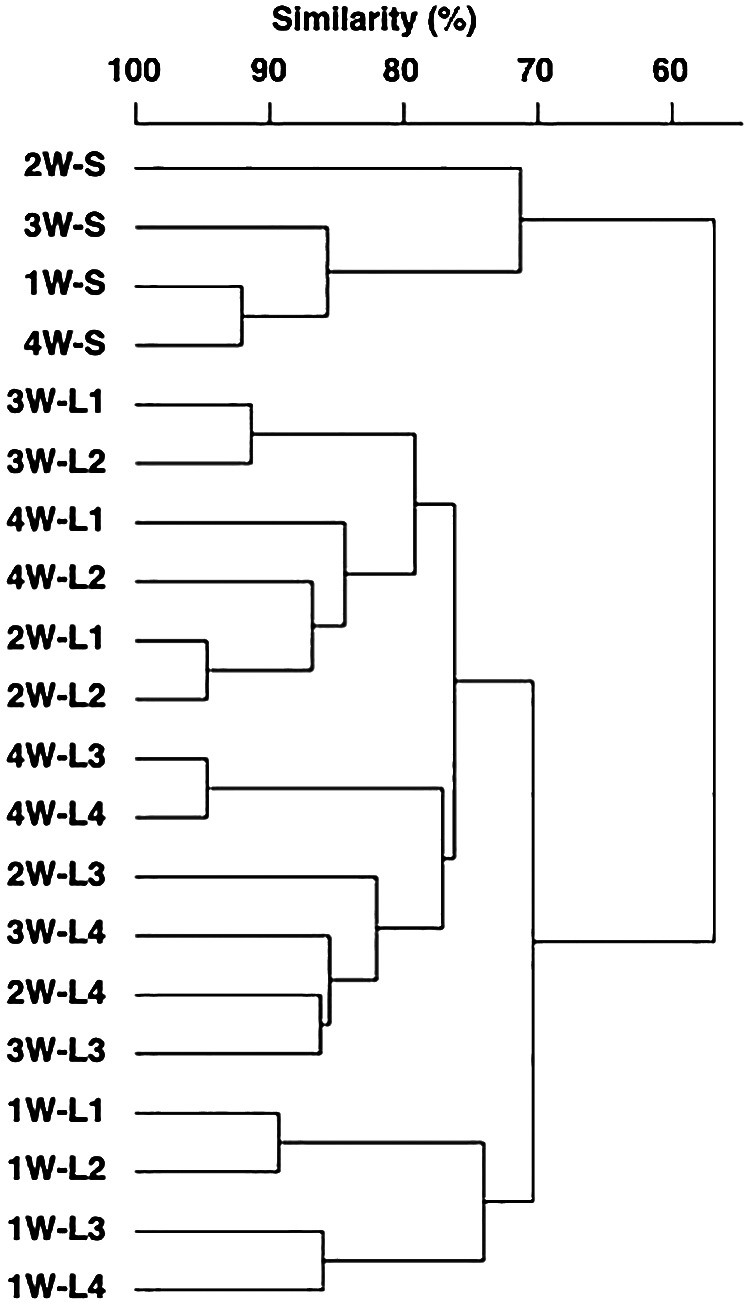
Dendrogram of microbial assemblages in four layers of the water column and surface sediment during the monitoring period. In the label, the number before “W” indicates the week in which sampling was conducted, and the number after “L” indicates the layer that was sampled; “S” refers to sediment.

The microbial assemblage composition during the monitoring period markedly differed between the water column and sediment (Fig. 2). Furthermore, the assemblage composition within the water column differed between the first week and other weeks, whereas that within sediment differed between the second week and other weeks. Since the assemblage composition and its temporal variations differed between the water column and sediment, the environmental factors involved in changes in the microbial assemblage were analyzed separately in the following sections.

### Environmental factors affecting microbial assemblages in the water column

**Fig. 3.**
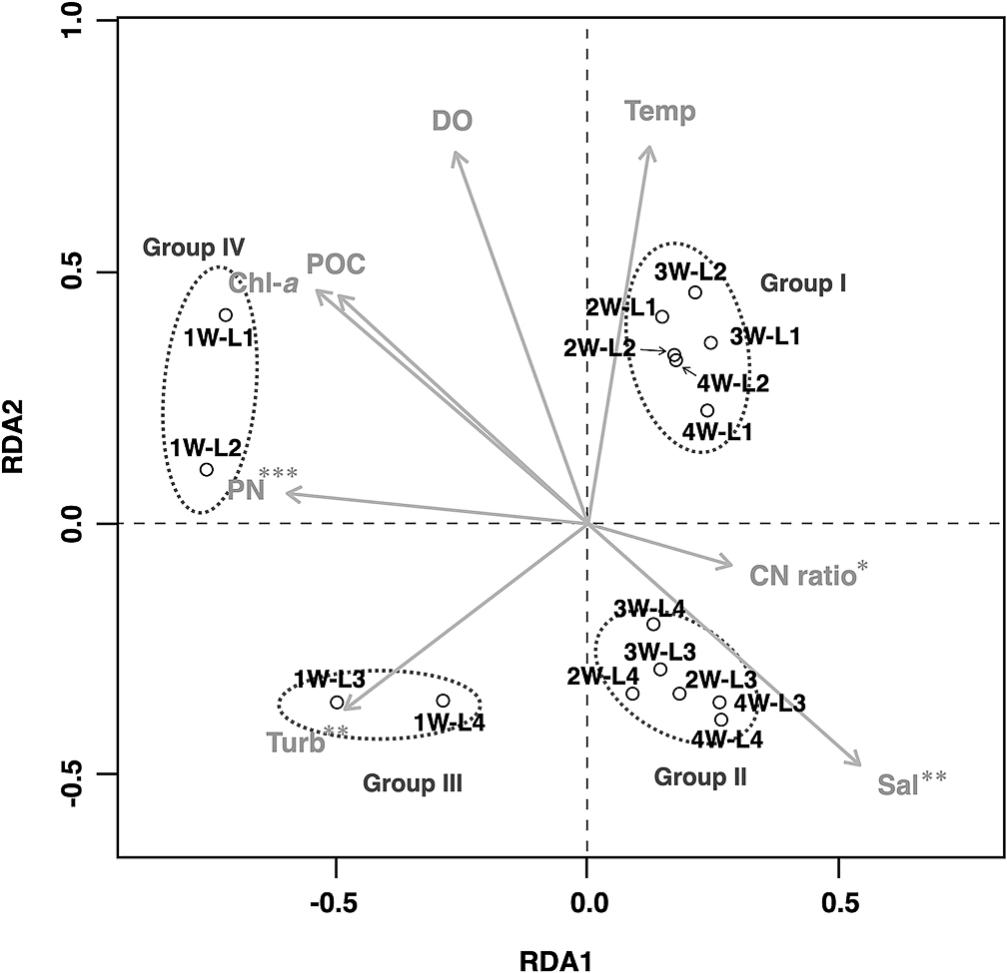
RDA ordination plots of the relationship between microbial assemblages and environmental factors. For an explanation of the labels indicating the microbial assemblages in the figure, refer to the legend to Fig. 2. Environmental factors that were significant in the Monte Carlo permutation test (999 randomized runs) are marked with an asterisk (***: *P*<0.001, **: *P*<0.01, *: *P*<0.05). Abbreviations: Temp, water temperature; Sal, salinity; Turb, turbidity.

The microbial assemblages in the four layers of the water column during the monitoring period were divided into four groups by RDA (Fig. 3). The classified assemblage groups (Group I: 2W-L1, 2W-L2, 3W-L1, 3W-L2, 4W-L1, and 4W-L2; Group II: 2W-L3, 2W-L4, 3W-L3, 3W-L4, 4W-L3, and 4W-L4; Group III: 1W-L1 and 1W-L2; and Group IV: 1W-L3 and 1W-L4) showed significantly different compositions among the groups (PERMANOVA, *P*<0.001), which was consistent with the results of the cluster ana­lysis (Fig. S3). The top three dominant OTUs in each group and their compositions were as follows: *Synechococcus* (30.0%), *Rhodobacteraceae* (8.0%), *Pelagibacter ubique* (7.2%), and other *Pelagibacteraceae* (7.2%) in Group I; *Rhodobacteraceae* (13.7%), *OCS155* (9.7%), and *Flavobacteriaceae* (9.0%) in Group II; *Cyclobacteriaceae* (14.4%), *Stramenopiles* (13.9%), and *Rhodobacteraceae* (12.0%) in Group III; and *Cyclobacteriaceae* (41.7%), *Synechococcus* (14.6%), and *Candidatus Aquiluna rubra* (8.2%) in Group IV (Table 1).

The environmental factors affecting the composition of microbial assemblages in the water column were evaluated using temperature, salinity, DO, turbidity, Chl-*a*, POC, PN, and the CN ratio of POM (Table S1). RDA outputs showed that four variables significantly accounted for the differences observed among the four assemblage groups, namely, PN, turbidity, salinity, and the CN ratio of POM (Monte Carlo permutation tests, *P*<0.001, *P*<0.01, *P*<0.01, and *P*<0.05, respectively). Groups I and II presented environmental characteristics with higher CN ratios of POM and lower PN than Groups III and IV. Furthermore, Groups I and IV showed lower salinity and turbidity than Groups II and III.

### Environmental factors affecting microbial assemblages in surface sediment

**Fig. 4.**
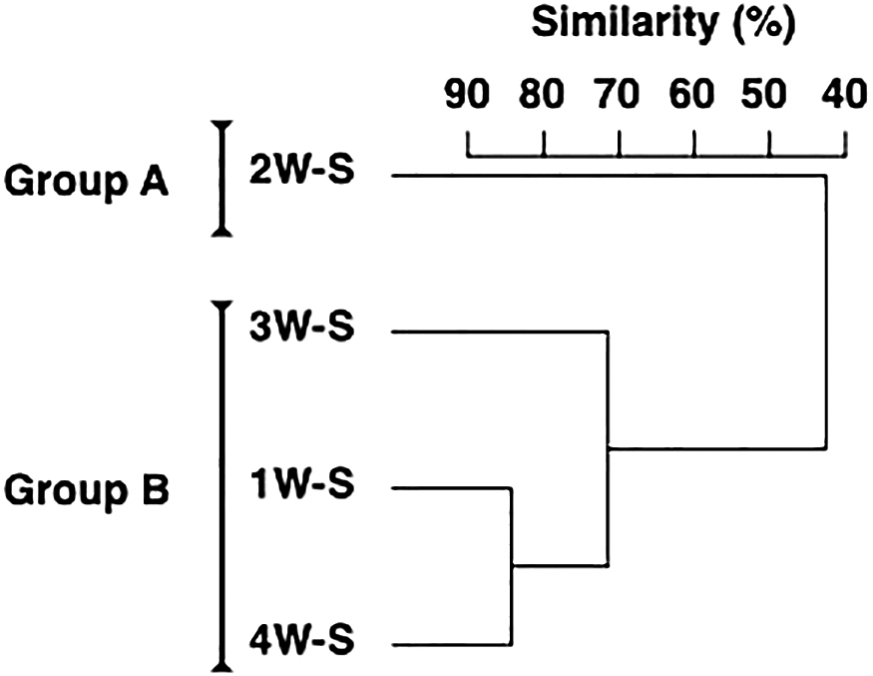
Dendrogram of microbial assemblages in surface sediment during the monitoring period.

The microbial assemblage in surface sediment was divided into two groups by a cluster ana­lysis: Group A consisting of 2W-S and Group B consisting of 1W-S, 3W-S, and 4W-S (Fig. 4). The top three dominant OTUs in each group and their compositions were as follows: *Sulfurimonas* (7.6%), *Desulfuromonadaceae* (7.6%), and *GCA004* (5.0%) in Group A; and *Piscirickettsiaceae* (11.8%), *Desulfobulbaceae* (7.0%), and *Desulfococcus* (5.1%) in Group B (Table 2). The differences observed between microbial assemblage groups were due to decreases in *Piscirickettsiaceae* and *Desulfobulbaceae* (contributions: 7.1 and 3.6%, respectively) and increases in *Sulfurimonas*, *Desulfuromonadaceae*, and *Fusibacter* (contributions: 6.6, 5.9, and 3.8%, respectively) in Group A from those in Group B.

**Fig. 5.**
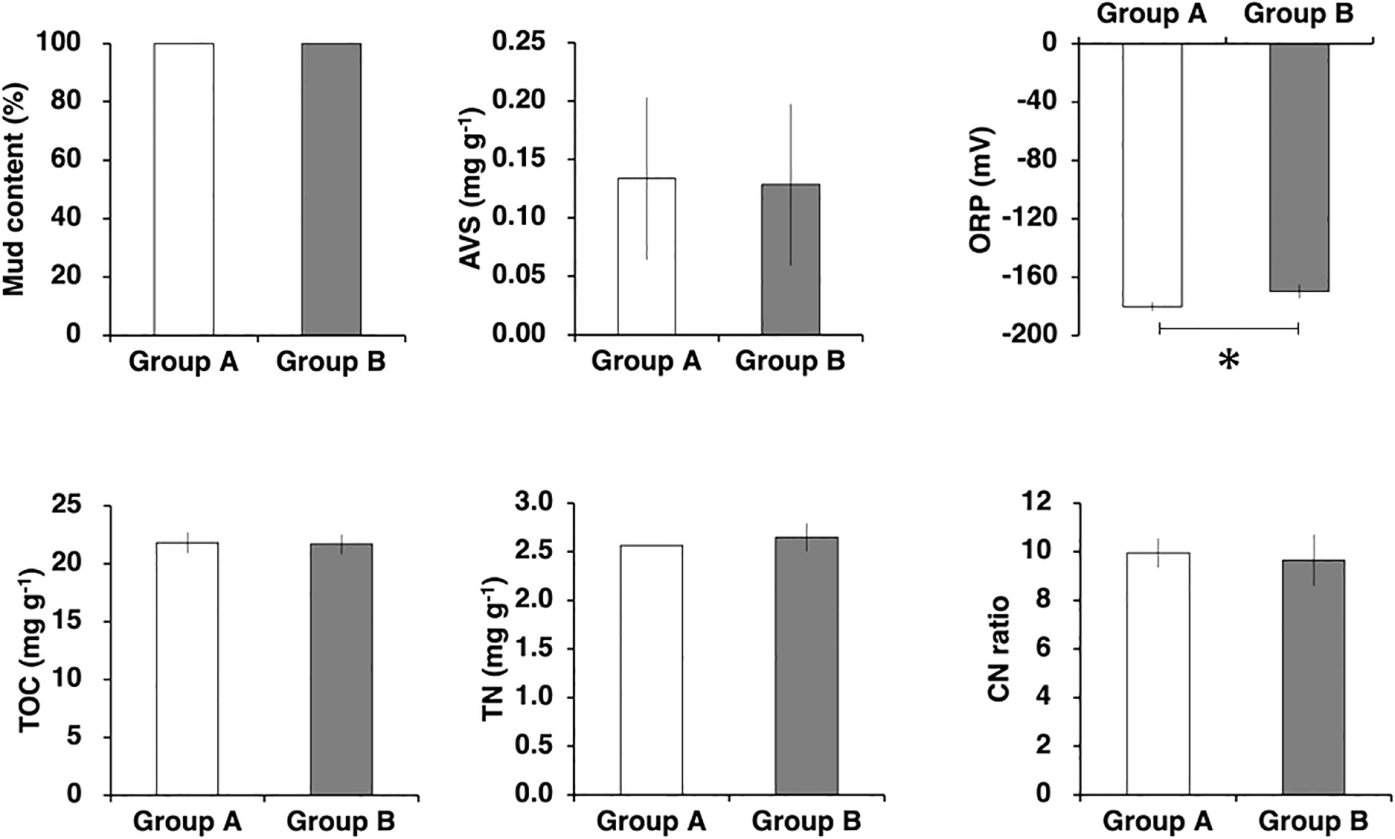
Comparison of mean environmental factors between two microbial assemblage groups in surface sediment, categorized by a cluster ana­lysis. The asterisk indicates a significant difference between groups (Wilcoxon rank-sum test,* P*<0.05).

To examine the environmental factors corresponding to the two sediment microbial assemblage groups divided by the cluster ana­lysis, the environmental factors of surface sediment were compared between the groups (Fig. 5). Only the ORP significantly differed between microbial assemblage groups among the environmental factors of surface sediment, with mean values of –180.0 and –169.8 mV for Groups A and B, respectively (Wilcoxon rank-sum test, *P*<0.05).

## Discussion

### Relationship between microbial assemblages in the water column and sediment

The present study demonstrated water-sediment coupling in shallow turbid water, inferred from the co-occurrence of ~70% of microorganisms, found both in the water column and in surface sediment, using a high-throughput sequence ana­lysis (Fig. 1). Although a few studies reported the proportion of OTUs shared between communities in water and those in sediment, a meta-ana­lysis revealed that communities in coastal waters shared an average of only 7.2% OTUs with the communities in sediment (Zinger *et al.*, 2011). In addition, a study conducted on the Yellow River, which is a highly turbid river, also found low numbers of shared OTUs between the communities in water and those in sediment (Xia *et al.*, 2014). The high percentage of OTUs commonly found in water and sediment samples in the present study may be unique to the estuaries at which vertical mixing frequently occurs with large tides. While the proportion of OTUs present in water and sediment was high, the composition and diversity of microorganisms differed between these two environments (Fig. 1 and 2). Multiple studies have reported this difference, and diversity in the microbial community in some cases was greater in sediment than in water (*e.g.*, coastal water [Zinger *et al.*, 2011], an estuary [Feng *et al.*, 2009; Zhang *et al.*, 2014; Wei *et al.*, 2016], hot spring [Cole *et al.*, 2013], lake [Ye *et al.*, 2009], and river [Staley *et al.*, 2015]), while the opposite was shown in other cases (*e.g.*, a river [Xia *et al.*, 2014] and open sea [Walsh *et al.*, 2016]). The present study detected higher OTU numbers in sediment than in the water column, which is consistent with the findings of previous studies conducted on coastal water and estuaries.

### Environmental factors affecting microbial assemblages in the water column

The first axis of the RDA reflected differences in sampling dates, namely, the first week and following weeks (Fig. 3). Between July 6 and 7, 2018, one week before sampling, daily mean flow from the Chikugo River, the largest river flowing into Ariake Bay, reached 2589.1~3887.5‍ ‍m^3^‍ ‍s^–1^ due to heavy rainfall (Ministry of Land, Infrastructure, Transport and Tourism, 2021). On day 1 of sampling, flow was recorded at 172.1 m^3^ s^–1^, the highest during the sampling period (after the second sampling, flow ranged between 52.4 and 106.9 m^3^ s^–1^). Therefore, the influence of this runoff may have been greater during the first week of sampling. The aquatic environment at this time showed higher turbidity in the water column and lower salinity as well as higher Chl-*a* in the surface layers than in the other sampling weeks (Table S1 and Fig. S4). In addition, the amounts of POC and PN in each layer in the water column were high in the first week (Table S1), and the CN ratio of POM suggested that a large amount of phytoplankton-derived organic matter was produced (Meyers, 1994). The microbial assemblage groups in the first week corresponded to Groups III and IV, which were both dominated by *Cyclobacteriaceae* (Table 1). Although this bacterial family is very versatile, some of its species are known to markedly increase their biomass after algal blooms (Eckert *et al.*, 2012; Fu *et al.*, 2020). During the sampling period, the occurrence of bloom by diatoms (taxa [maximum cell density]: *Skeletonema* spp. [167,600 cells mL^–1^], *Thalassiosira* spp. [1,480 cells mL^–1^],* Chaetoceros* spp. [4,490 cells mL^–1^]) was reported in the study area (Kyushu Fisheries Coordinate Office, 2018). Therefore, *Cyclobacteriaceae* in this study may be copiotrophic when organic matter is supplied from autotrophic phytoplankton.

The second axis of the RDA reflected the difference in water depth, with positive and negative values indicating the upper and lower two layers, respectively (Fig. 3). On all sampling days, the thermocline and halocline were observed at a depth of between 3 and 5‍ ‍m (Fig. S4); therefore, the upper and lower two layers in the present study represented different water mass environments within the water column. The surface layer presented lower salinity, a higher water temperature, and a higher DO environment than the bottom layer (Fig. 3 and Table S1). The microbial assemblage group on the surface (Group I) was dominated by *Synechococcus* and *Rhodobacteraceae* (as well as *Pelagibacter ubique*), which may partially include light-related bacteria. The genus *Synechococcus* comprises autotrophic cyanobacteria; therefore, species belonging to this genus were predominant in the two surface layers for oxygenic photosynthesis. Moreover, some *Rhodobactericeae* (*e.g.*, *Rhodobacter* spp.) contain bacteriochlo­rophylls for their aerobic anoxygenic photosynthetic activity (Deisenhofer *et al.*, 1985; Pujalte *et al.*, 2014; Pohlner *et al.*, 2019). In addition, *Synechococcus* negatively correlated with salinity (Table S3). The capacity of *Synechococcus* to adapt to changes in salinity was previously reported to be high (Kim *et al.*, 2018), and this characteristic may also be a reason for its dominance in low-salinity surface layers. A member of the most commonly distributed bacteria *SAR11*, *Pelagibacter*, particularly *P. ubique*, may also utilize light energy via proteorhodopsin (Steindler *et al.*, 2011) for proton pumping and the consequent generation of ATP (Béjá *et al.*, 2001; Sabehi *et al.*, 2005). On the other hand, Group II, corresponding to the microbial assemblage group of the bottom layer, was dominated by *Rhodobacteraceae* and *OCS155* as well as *Flavobacteriaceae*. *Rhodobactraceae* were predominant in the bottom layers, while also being abundant in the sun-lit upper layers. This microbial family has a highly diverse physiology and ecology (Pujalte *et al.*, 2014), and the species found in the bottom layers may have either fallen to the bottom from the upper layer or may have actively grown by switching to heterotrophy. *Flavobacteriaceae* and *OCS155* were previously reported to proliferate with a supply of organic detritus (*e.g.*, senescent/dead algal cells) (Bernardet and Bowman, 2006; Alonso *et al.*, 2007; Mann *et al.*, 2013; Chen *et al.*, 2016; Easson and Lopez, 2019), indicating that heterotrophic bacteria were dominant in the light-limited bottom layer.

### Environmental factors affecting microbial assemblages in surface sediment

*Sulfurimonas*, *Desulfuromonadaceae*, *Piscirickettsiaceae*, and *Desulfobulbaceae* were mostly found in the sediment (Table 1 and 2). Among them, *Sulfurimonas* oxidize sulfur compounds, while *Desulfuromonadaceae* and *Desulfobulbaceae* reduce them and, together, these bacterial species are involved in sulfur cycling in sediment (Rodriguez-Mora *et al.*, 2015; Suter *et al.*, 2018; see also van Vliet *et al.*, 2021). The composition of bacteria involved in the sulfur cycle has been reported to vary with salinity (Kondo *et al.*, 2007). In the present study, changes in salinity were observed in the surface layer of the water column during the sampling period, while few changes were noted in salinity in the bottom waters (Table S1), suggesting that changes in the bacterial assemblages involved in the sulfur cycle were caused by other factors. Sulfur oxidation/reduction generally indicates a lack of major electron donors (*i.e.*, oxygen and manganese); therefore, the sulfur cycle is activated by the redox state in the sediment becoming more reductive (Emerson and Hedges, 1988; Reimers *et al.*, 1992; Takii *et al.*, 2002; Sarmiento and Gruber, 2006). Since the DO of the bottom water during the sampling period was hypoxic (Table S1), sulfur-related bacteria may have been dominant in surface sediment. Furthermore, increases in bacteria associated with sulfur cycling contributed to the difference in the sediment assemblage composition in the second week when the ORP was more reduced (Fig. 4 and 5). Microbial assemblages in surface sediment were similar between the first and fourth weeks, indicating the process of gradual recovery from the changed composition in the second week to the original composition of the microbial assemblage (Fig. 4). The results of the present study indicate that changes in the redox environment markedly affected microbial communities in estuarine sediment, and that changes in microbial compositions in surface sediment, which were associated with the reduction of bottom sediment, occurred in less than one week, while changes in the microbial composition after recovery from this reduction may take approximately two weeks.

## Conclusions

In the present study, weekly field surveys were conducted on four layers corresponding to different water depths and sediment surfaces to examine the relationship between microbial assemblages in the water column and sediment surface and identify the environmental factors that influence the short-term dynamics of microbial assemblages in these two zones. Although the results obtained suggested water–sediment coupling, as inferred from the high proportion of OTUs present in both water and sediment (Fig. 1), the composition of microbial assemblages between the two zones differed even in highly turbid brackish waters with high sediment resuspension (Fig. 2). In the water column, microbial assemblages changed in response to the different environmental factors in water before and after heavy rainfall runoff, and in water above and below the thermocline and halocline (Fig. 3). On the other hand, microbial assemblages in sediment were altered by changes in the redox environment (Fig. 5). The microbial assemblages in the water column and sediment surface in the inner part of Ariake Bay during summer shows that the timing of changes in the microbial composition and the environmental factors associated with these changes were different.

## Citation

Orita, R., Yoshida, K., Terazono, H., Nagano, Y., Goto, M., Kimura, K., and Kobayashi, G. (2022) Weekly Observations of Estuarine Microbial Assemblages during Summer in the Inner Part of Ariake Bay, Japan; Microbial Water-sediment Coupling in Turbid Shallow Waters. *Microbes Environ ***37**: ME22015.

https://doi.org/10.1264/jsme2.ME22015

## Supplementary Material

Supplementary Material

## Figures and Tables

**Table 1.** Dominant OTUs of four microbial groups in the water column classified by RDA.

Rank	Group I				Group II				Group III				Group IV		
	OTUs	RC^a^	CC^b^		OTUs	RC^a^	CC^b^		OTUs	RC^a^	CC^b^		OTUs	RC^a^	CC^b^
1	*Synechococcus*	30.0	30.0		*Rhodobacteraceae*	13.7	13.7		*Cyclobacteriaceae*	14.4	14.4		*Cyclobacteriaceae*	41.7	41.7
2	*Rhodobacteraceae*	8.0	38.0		*OCS155*	9.7	23.4		*Stramenopiles*	13.9	28.3		*Synechococcus*	14.6	56.3
3	*Pelagibacter ubique*	7.2	45.2		*Flavobacteriaceae*	9.0	32.4		*Rhodobacteraceae*	12.0	40.3		*Candidatus Aquiluna rubra*	8.2	64.5
4	other *Pelagibacteraceae*	7.2	52.4		*Synechococcus*	8.3	40.7		*Flavobacteriaceae*	8.3	48.6		*Rhodobacteraceae*	7.0	71.5
5	*Stramenopiles*	5.7	58.1		other *Pelagibacteraceae*	4.2	44.9		*Pelagibacter ubique*	5.0	53.6		*Flavobacteriaceae*	6.7	78.2
6	*Flavobacteriaceae*	5.2	63.3		*Cryomorphaceae*	4.0	48.9		*OCS155*	4.3	57.9		*Stramenopiles*	4.0	82.2
7	*OCS155*	4.6	67.9		*Pelagibacter ubique*	3.7	52.6		*Synechococcus*	3.6	61.5		*Cryomorphaceae*	2.9	85.1
8	*Cryomorphaceae*	3.3	71.2		*Balneola*	3.3	55.9		*Nitrosopumilus*	3.0	64.5		*Fluviicola*	2.3	87.4
9	*Balneola*	2.4	73.6		*Coccinimonas marina*	2.6	58.5		*Ruegeria*	2.5	67.0		*Altererythrobacter ishigakiensis*	0.9	88.3
10	*Mamiellaceae*	2.1	75.7		*Tenacibaculum*	2.3	60.8		*Cryomorphaceae*	2.2	69.2		*Algoriphagus aquatilis*	0.8	89.1
	Others	24.3	100.0		Others	39.2	100.0		Others	30.8	100.0		Others	10.9	100.0

^a^ Relative composition (%) ^b^ Cumulative composition (%)

**Table 2.** Dominant OTUs of two sediment microbial groups classified by a cluster ana­lysis. The main OTUs contributing to differences between groups were evaluated using SIMPER.

Rank	Group A				Group B				Influential OTUs	Contr. (%)
	OTUs	RC^a^	CC^b^		OTUs	RC^a^	CC^b^			
1	*Sulfurimonas*	7.6	7.6		*Piscirickettsiaceae*	11.8	11.8		*Piscirickettsiaceae*	7.1
2	*Desulfuromonadaceae*	7.6	15.2		*Desulfobulbaceae*	7.0	18.8		*Sulfurimonas*	6.6
3	*GCA004*	5.0	20.2		*Desulfococcus*	5.1	23.9		*Desulfuromonadaceae*	5.9
4	*Fusibacter*	4.3	24.5		*Marinicellaceae*	4.7	28.6		*Fusibacter*	3.8
5	*Helicobacteraceae*	4.0	28.5		*GCA004*	3.5	32.1		*Desulfobulbaceae*	3.6
6	*Photobacterium*	3.9	32.4		other *Gammaproteobacteria*	3.2	35.3		*Helicobacteraceae*	3.4
7	*Piscirickettsiaceae*	3.6	36.0		*Stramenopiles*	3.2	38.5		*Photobacterium*	3.4
8	other* Bacteroidetes*	3.5	39.5		*Chromatiales*	3.0	41.5		*Desulfococcus*	2.9
9	*Bacteroidales*	3.2	42.7		other *Deltaproteobacteria*	2.5	44.0		other *Bacteroidetes*	2.7
10	*Desulfobulbaceae*	2.8	45.5		*OS-K*	2.4	46.4		*Stramenopiles*	2.6
	Others	54.5	100.0		Others	53.6	100.0		Total	42.0

^a^ Relative composition (%) ^b^ Cumulative composition (%)

## References

[B1] Alonso, C., Warnecke, F., Amann, R., and Pernthaler, J. (2007) High local and global diversity of *Flavobacteria* in marine plankton. Environ Microbiol 9: 1253–1266. 17472638 10.1111/j.1462-2920.2007.01244.x

[B2] Anderson, M.J. (2001) A new method for non-parametric multivariate ana­lysis of variance. Austral Ecol 26: 32–46.

[B3] Baker, B.J., Appler, K.E., and Gong, X. (2012) New microbial biodiversity in marine sediments. Ann Rev Mar Sci 13: 161–175. 10.1146/annurev-marine-032020-014552 32746696

[B4] Béjá, O., Spundich, E.N., Spundich, J.L., Lecierc, M., and DeLong, E.F. (2001) Proteorhodopsin phototrophy in the ocean. Nature 411: 786–789. 11459054 10.1038/35081051

[B5] Bernardet, J.F., and Bowman, J.P. (2006) The Genus *Flavobacterium*. In *The Prokaryotes*, 3rd edn. Dworkin, M., Falkow, S., Rosenberg, E., Schleifer, K.H., and Stackebrandt, E. (eds). New York, NY: Springer, pp. 481–531.

[B6] Bolger, A.M., Lohse, M., and Usadel, B. (2014) Trimmomatic: A flexible trimmer for Illumina sequence data. Bioinformatics 30: 2114–2120. 24695404 10.1093/bioinformatics/btu170 PMC4103590

[B7] Bray, J.R., and Curtis, J.T. (1957) An ordination of the upland forest communities of southern Wisconsin. Ecol Monogr 27: 325–349.

[B8] Chen, H., Zhang, H., Xiong, J., Wang, K., Zhu, J., Zhu, X., et al. (2016) Successional trajectories of bacterioplankton community over the complete cycle of a sudden phytoplankton bloom in the Xiangshan Bay, East China Sea. Environ Pollut 219: 750–759. 27453358 10.1016/j.envpol.2016.07.035

[B9] Cole, J.K., Peacock, J.P., Dodsworth, J.A., Williams, A.J., Thompson, D.B., Dong, H., et al. (2013) Sediment microbial communities in Great Boiling Spring are controlled by temperature and distinct from water communities. ISME J 7: 718–729. 23235293 10.1038/ismej.2012.157 PMC3605714

[B10] Curtis, T.P., Sloan, W.T., and Scanneell, J.W. (2002) Estimating prokaryotic diversity and its limits. Proc Natl Acad Sci U S A 99: 10494–10499. 12097644 10.1073/pnas.142680199 PMC124953

[B11] Deisenhofer, J., Epp, O., Huber, M.R., and Michel, H. (1985) Structure of the protein subunits in the photosynthetic reaction centre of *Rhodopseudomonas viridis* at 3Å resolution. Nature 318: 618–624. 22439175 10.1038/318618a0

[B12] Easson, C.G., and Lopez, J.V. (2019) Depth-dependent environmental drivers of microbial plankton community structure in the northern Gulf of Mexico. Front Microbiol 9: 3175. 30662434 10.3389/fmicb.2018.03175 PMC6328475

[B13] Eckert, E.M., Salcher, M.M., Posch, T., Eugster, B., and Pernthaler, J. (2012) Rapid successions affect microbial *N*-acetyl-glucosamine uptake patterns during a lacustrine spring phytoplankton bloom. Environ Microbiol 14: 794–806. 22082109 10.1111/j.1462-2920.2011.02639.x

[B14] Emerson, S., and Hedges, J.I. (1988) Processes controlling the organic carbon content of open ocean sediments. Paleoceanogr Paleoclimatol 3: 621–634.

[B15] Feng, B.W., Li, X.R., Wang, J.H., Hu, Z.Y., Meng, H., Xiang, L.Y., and Quan, Z.X. (2009) Bacterial diversity of water and sediment in the Changjiang estuary and coastal area of the East China Sea. FEMS Microbiol Ecol 70: 236–248. 10.1111/j.1574-6941.2009.00772.x 19780829

[B16] Fu, H., Smith, C.B., Sharma, S., and Moran, M.A. (2020) Genome sequences and metagenome-assembled genome sequences of microbial communities enriched on phytoplankton exometabolites. Microbiol Resour Announce 9: e00724-20. 10.1128/MRA.00724-20 PMC7378039 32703840

[B17] Hayami, Y., Wada, M., Umezawa, Y., Fujii, N., Nakamura, A., and Mori, F. (2019) Hypoxic water mass in the highly turbid well-mixed macrotidal Rokkaku River Estuary, Ariake Sea, Japan. Estuarine, Coastal Shelf Sci 219: 210–222.

[B18] Herlemann, D.P., Labrenz, M., Jürgens, K., Bertilsson, S., Waniek, J.J., and Andersson, A.F. (2011) Transitions in bacterial communities along the 2000‍ ‍km salinity gradient of the Baltic Sea. ISME J 5: 1571–1579. 21472016 10.1038/ismej.2011.41 PMC3176514

[B19] Kaku, N., Ueki, A., Ueki, K., and Watanabe, K. (2005) Methanogenesis as an important terminal electron accepting process in estuarine sediment at the mouth of Orikasa river. Microbes Environ 20: 41–52.

[B20] Khandeparker, L., Kuchi, N., Kale, D., and Anil, A.C. (2017) Microbial community structure of surface sediments from a tropical estuarine environment using next generation sequencing. Ecol Indic 74: 172–181.

[B21] Kim, Y., Jeon, J., Kwak, M.S., Kim, G.H., Koh, I., and Rho, M. (2018) Photosynthetic functions of *Synechococcus* in the ocean microbiomes of diverse salinity and seasons. PLoS One 13: e0190266. 29293601 10.1371/journal.pone.0190266 PMC5749766

[B22] Kirchman, D.L. (2012) *Processes in Microbial Ecology*. Oxford, UK: Oxford University Press.

[B23] Koh, C.H., Khim, J.S., Araki, H., Yamanishi, H., Mogi, H., and Koga, K. (2006) Tidal resuspension of microphytobenthic chlo­rophyll *a* in a Nanaura mudflat, Saga, Ariake Sea, Japan: flood–ebb and spring–neap variations. Mar Ecol Prog Ser 312: 85–100.

[B24] Kondo, R., Purdy, K.J., de Queiroz Silva, S., and Nedwell, D.B. (2007) Spatial dynamics of sulphate-reducing bacterial compositions in sediment along a salinity gradient in a UK estuary. Microbes Environ 22: 11–19.

[B25] Kyushu Fisheries Coordinate Office. (2018) Red Tides in the Coastal Waters of Kyushu. URL https://www.jfa.maff.go.jp/kyusyu/sigen/attach/pdf/akashio_kyusyu-10.pdf

[B26] Legendre, P., and Gallagher, E.D. (2001) Ecologically meaningful transformations for ordination of species data. Oecologia 129: 271–280. 28547606 10.1007/s004420100716

[B27] Mahmoudi, N., Robeson, M.S., Castro, H.F., Fortney, J.L., Techtmann, S.M., Joyner, D.C. et al. (2015) Microbial community composition and diversity in Caspian Sea sediments. FEMS Microbiol Ecol 91: 1–11. 10.1093/femsec/fiu013 PMC4399438 25764536

[B28] Mann, A.J., Hahnke, R.L., Huang, S., Werner, J., Xing, P., Barbeyron, T., et al. (2013) The genome of the alga-associated marine Flavobacterium *Formosa agariphila* KMM 3901T reveals a broad potential for degradation of algal polysaccharides. Appl Environ Microbiol 79: 6813–6822. 23995932 10.1128/AEM.01937-13 PMC3811500

[B29] Meyers, P.A. (1994) Preservation of elemental and isotopic source identification of sedimentary organic matter. Chem Geol 114: 289–302.

[B30] Ministry of Land, Infrastructure, Transport and Tourism. (2021) Water Information System (in Japanese). URL http://www1.river.go.jp/

[B31] Orita, R., Umehara, A., Komorita, T., Choi, J.W., Montani, S., Komatsu, T., and Tsutsumi, H. (2015) Contribution of the development of the stratification of water to the expansion of dead zone: a sedimentological approach. Estuarine, Coastal Shelf Sci 164: 204–213.

[B32] Pohlner, M., Dlugosch, L., Wemheuer, B., Milis, H., Engelen, B., and Reese, B.R. (2019) The majority of active *Rhodobacteraceae* in marine sediments belong to uncultured genera: a molecular approach to link their distribution to environmental conditions. Front Microbiol 10: 659. 31001232 10.3389/fmicb.2019.00659 PMC6454203

[B33] Pujalte, M.J., Lucena, T., Ruvira, M.A., Arahal, D.A., and Macián, M.C. (2014) The Family *Rhodobacteraceae*. In *The Prokaryotes: Alphaproteobacteria* and *Betaproteobacteria*. Rosenberg, E., DeLong, E.F., Lory, S., Stackebrandt, E., and Thompson, F. (eds). Berlin, Heidelberg: Springer, pp. 439–512.

[B34] Qian, G., Wang, J., Kan, J., Zhang, X., Xia, Z., Zhang, X., et al. (2018) Diversity and distribution of anammox bacteria in water column and sediments of the Eastern Indian Ocean. Int Biodeterior Biodegrad 133: 52–62.

[B35] Reimers, C.E., Jahnke, R.A., and McCorkle, D.C. (1992) Carbon fluxes and burial rates over continental slope and rise off central California with implications for the global carbon cycle. Global Biogeochem Cycles 6: 199–224.

[B36] Rodriguez-Mora, M.J., Scranton, M.I., Taylor, G.T., and Chistoserdov, A.Y. (2015) The dynamics of the bacterial diversity in the redox transition and anoxic zones of the Cariaco Basin assessed by parallel tag sequencing. FEMS Microbiol Ecol 91: fiv088. 26209697 10.1093/femsec/fiv088

[B37] Sabehi, G., Loy, A., Jung, K.H., Partha, R., Spudich, J.L., Isaacson, T., et al. (2005) New insights into metabolic properties of marine bacteria encoding proteorhodopsins. PLoS Biol 8: e273. 10.1371/journal.pbio.0030273 PMC1175822 16008504

[B38] Sakami, T. (2012) Distribution of ammonia-oxidizing archaea and bacteria in the surface sediments of Matsushima Bay in relation to environmental variables. Microbes Environ 27: 61–66. 22200641 10.1264/jsme2.ME11218 PMC4036025

[B39] Sarmiento, J.L., and Gruber, N. (2006) *Ocean Biogeochemical Dynamics*. Princeton, NJ: Princeton University Press.

[B40] Sato, M. (2010) Anthropogenic decline of the peculiar fauna of estuarine mudflats in Japan. Plankton Benthos Res 5: 202–213.

[B41] Staley, C., Gould, T.J., Wang, P., Phillips, J., Cotner, J.B., and Sadowsky, M.J. (2015) Species sorting and seasonal dynamics primarily shape bacterial communities in the Upper Mississippi River. Sci Total Environ 505: 435–445. 25461045 10.1016/j.scitotenv.2014.10.012

[B42] Steindler, L., Schwalbach, M.S., Smith, D.P., Chan, F., and Giovannoni, S.J. (2011) Energy starved *Candidatus* Pelagibacter Ubique substitutes light-mediated ATP production for endogenous carbon respiration. PLoS One 6: e19725. 21573025 10.1371/journal.pone.0019725 PMC3090418

[B43] Suter, E.A., Pachiadaki, M., Taylor, G.T., Astor, Y., and Edgcomb, V.P. (2018) Free-living chemoautotrophic and particle-attached heterotrophic prokaryotes dominate microbial assemblages along a pelagic redox gradient. Environ Microbiol 20: 693–712. 29160034 10.1111/1462-2920.13997

[B44] Takii, S., Tanaka, H., Kohata, K., Nakamura, Y., Ogura, H., and Takeshita, S. (2002) Seasonal changes in sulfate reduction in sediments in the inner part of Tokyo Bay. Microbes Environ 17: 10–17.

[B45] Tanaka, K., and Kodama, M. (2007) Effects of resuspended sediments on the environmental changes in the inner part of Ariake Bay, Japan. Bull Fish Res Agency 19: 9–15.

[B46] Tsutsumi, H. (2006) Critical events in the Ariake Bay ecosystem: clam population collapse, red tides, and hypoxic bottom water. Plankton Benthos Res 1: 3–25.

[B47] van Vliet, D.M., von Meijenfeldt, F.A.B., Dutilh, B.A., Villanueva, L., Damsté, J.S.S., Stams, A.J.M., and Sánchez-Andrea, I. (2021) The bacterial sulfur cycle in expanding dysoxic and euxinic marine waters. Environ Microbiol 23: 2834–2857. 33000514 10.1111/1462-2920.15265 PMC8359478

[B55] Walsh, E.A., Kirkpatrick, J.B., Rutherford, S.D., Smith, D.C., Sogin, M., and D’Hondt, S. (2016) Bacterial diversity and community composition from seasurface to subseafloor. ISME J 10: 979–989. 26430855 10.1038/ismej.2015.175 PMC4796937

[B48] Wei, G., Li, M., Li, F., Li, H., and Gao, Z. (2016) Distinct distribution patterns of prokaryotes between sediment and water in the Yellow River estuary. Appl Microbiol Biotechnol 100: 9683–9697. 27557722 10.1007/s00253-016-7802-3

[B49] Whitman, W.B., Coleman, D.C., and Wiebe, W.J. (1998) Prokaryotes: the unseen majority. Proc Natl Acad Sci U S A 95: 6578–6583. 9618454 10.1073/pnas.95.12.6578 PMC33863

[B50] Xia, N., Xia, X., Liu, T., Hu, L., Zhu, B., Zhang, X., and Dong, J. (2014) Characteristics of bacterial community in the water and surface sediment of the Yellow River, China, the largest turbid river in the world. J Soils Sediments 14: 1894–1904.

[B51] Yanagi, T., and Shimomura, M. (2006) Seasonal variation in the transverse and layered structure of estuarine circulation in Ariake Bay, Japan. Cont Shelf Res 26: 2598–2606.

[B52] Ye, W., Liu, X., Lin, S., Tan, J., Pan, J., Li, D., and Yang, H. (2009) The vertical distribution of bacterial and archaeal communities in the water and sediment of Lake Taihu. FEMS Microbiol Ecol 70: 263–276. 10.1111/j.1574-6941.2009.00761.x 19744240

[B53] Zhang, W., Bougouffa, S., Wang, Y., Lee, O.O., Yang, J., Chan, C., et al. (2014) Toward understanding the dynamics of microbial communities in an estuarine system. PLoS One 9: e94449. 24732211 10.1371/journal.pone.0094449 PMC3986090

[B54] Zinger, L., Amaral-Zettler, L.A., Fuhrman, J.A., Horner-Devine, M.C., Huse, S.M., Welch, D.B.M., et al. (2011) Global patterns of bacterial beta-diversity in seafloor and seawater ecosystems. PLoS One 6: e24570. 21931760 10.1371/journal.pone.0024570 PMC3169623

