# Supplementary material for: Weekly Observations of Estuarine Microbial Assemblages during Summer in the Inner Part of Ariake Bay, Japan; Microbial Water-sediment Coupling in Turbid Shallow Waters
Source: Microbes Environ. 2022 Jun 8;37(2):ME22015. doi: 10.1264/jsme2.ME22015 (PMC9530734; doi:10.1264/jsme2.ME22015)
Supplement: Supplementary file 1 — Supplementary Material [file 37_22015_s1.pdf]

Table. S1 in Orita *et al*.

Table S1. The mean values of environmental factors at three replicate sites. For the explanation of sampling date and layer, see the legend to Figure 1.

Environmental factors	Layer	1st week	2nd week	3rd week	4th week
Temperature (℃)	1	29.0±0.6	28.8±0.3	29.9±0.3	29.1±0.1
	2	25.9±0.3	27.6±0.2	29.3±0.2	27.8±1.0
	3	23.6±0.0	24.2±0.0	24.1±0.0	25.5±0.0
	4	23.1±0.0	24.4±0.0	24.1±0.0	25.5±0.0
Salinity	1	14.6±0.6	26.2±0.1	26.1±0.1	28.4±0.0
	2	20.7±0.9	26.5±0.1	26.6±0.2	28.6±0.3
	3	29.7±0.1	28.9±0.0	30.2±0.0	29.9±0.0
	4	29.8±0.1	28.8±0.1	30.2±0.0	29.9±0.0
DO (mg/L)	1	14.3±0.5	8.0±0.3	9.5±0.1	9.5±0.2
	2	10.1±1.4	7.7±0.7	9.7±0.2	9.3±1.2
	3	2.9±0.1	2.0±0.1	2.0±0.1	2.9±0.1
	4	2.9±0.1	2.0±0.1	2.1±0.1	2.8±0.1
Turbidity	1	6.7±0.1	2.9±0.4	1.5±0.2	2.2±0.1
	2	4.1±0.4	2.9±0.5	1.7±0.1	2.7±0.5
	3	8.2±3.4	6.7±0.4	4.0±1.0	1.9±0.2
	4	11.2±1.7	4.6±1.7	4.6±1.7	2.0±0.1
Chlorophyll *a* (μg/L)	1	62.9±10.2	14.5±2.3	12.9±4.3	11.1±1.9
	2	48.0±14.4	21.6±6.3	23.3±1.8	18.6±3.7
	3	4.4±0.8	5.9±0.6	12.7±1.4	6.4±0.4
	4	4.9±0.5	7.0±0.5	13.2±2.6	6.6±0.9
POC (μg/L)	1	1320.8±198.3	682.6±6.1	699.1±240.4	863.2±503.6
	2	930.4±183.2	658.2±65.4	915.2±78.1	674.3±50.6
	3	583.2±214.2	451.8±101.2	672.0±34.9	500.3±24.8
	4	950.4	395.8±52.0	605.1±140.8	532.2±68.4
PN (μg/L)	1	284.6±51.8	170.4	101.3±21.4	74.3±44.3
	2	323.9±81.6	61.9	127.9±11.4	73.8±62.7
	3	141.3±53.5	158.5	53.6±45.1	172.5±130.3
	4	220.0±16.7	18.2	72.5±14.1	170.0±163.1
C:N	1	5.4±0.3	5.5	8.1±2.2	13.6±1.1
	2	3.5±0.9	12.1	8.4±2.1	16.5±6.4
	3	4.8±0.3	4.8±0.4	28.1±6.7	4.8±2.8
	4	4.8	25.8	10.1±3.7	6.3±4.1

Table. S2 in Orita *et al*.

Table S2. Microbial assemblage richness, diversity and evenness indices and coverage value. For the explanation of sampling date and layer, see the legend to Figure 1.

Samplig date	Samling layer	OTU richness	*H'* diversity[1]	*J'* evenness[2]	Coverage[3]
1st week	layer 1	110	2.98	0.44	0.93
1st week	layer 2	138	3.43	0.48	0.94
1st week	layer 3	219	4.75	0.61	1.00
1st week	layer 4	238	5.03	0.64	0.97
1st week	sediment	213	5.95	0.77	0.97
2nd week	layer 1	125	3.81	0.55	0.99
2nd week	layer 2	152	4.14	0.57	0.99
2nd week	layer 3	174	5.15	0.69	0.99
2nd week	layer 4	185	5.02	0.67	0.98
2nd week	sediment	247	5.99	0.75	1.00
3rd week	layer 1	125	4.35	0.62	0.96
3rd week	layer 2	101	4.21	0.63	0.99
3rd week	layer 3	125	4.96	0.71	0.99
3rd week	layer 4	170	5.26	0.71	0.99
3rd week	sediment	264	5.95	0.74	1.00
4th week	layer 1	156	4.20	0.58	0.98
4th week	layer 2	125	3.59	0.52	0.99
4th week	layer 3	130	4.45	0.63	0.98
4th week	layer 4	156	4.60	0.63	0.98
4th week	sediment	234	6.10	0.78	1.00

[1]: Shannon-Wiener diversity index (Calculated using R with the package 'vegan'.).

[2]: Pielou's evenness index ($J' = H'/\log_2 S$).

[3]: Good's coverage value (Coverage = $1 - (n_1/N)$, n_1: the number of singleton OTUs, N: the total number of OTUs).

Table. S3 in Orita *et al*.

Table S3. Correlations between dominant OTUs in the water column and environmental factors. For the dominant OTUs in the water column, see Table 1. The correlation coefficient is calculated using Spearman's rank correlation coefficient (***: *P* >0.001, **: *P* >0.01, *: *P* >0.05).

OTU	Temperature	Salinity	Chl.*a*	Trubidity	DO	POC	PN	C/N
Nitrosopumilus	-0.73 **	0.77 ***	-0.79 ***	0.34	-0.73 **	-0.44	-0.07	-0.09
OCS155	-0.42	0.74 **	-0.46	0.00	-0.73 **	-0.67 **	-0.60 **	0.48
Candidatus Aquiluna rubra	0.04	-0.40	0.45	0.37	0.39	0.56 *	0.25	-0.20
Cyclobacteriaceae	-0.43	-0.11	0.05	0.79 ***	0.06	0.32	0.20	-0.22
Algoriphagus aquatilis	-0.34	-0.18	0.16	0.61 *	0.28	0.38	0.41	-0.44
Cryomorphaceae	-0.06	0.44	0.09	-0.33	-0.23	-0.19	-0.33	0.38
Fluviicola	-0.09	0.02	0.17	0.16	0.25	0.23	0.27	-0.09
Flavobacteriaceae	-0.82 ***	0.69 **	-0.43	0.49	-0.80 ***	-0.69 **	-0.24	0.00
Coccinimonas marina	-0.04	0.47	-0.07	-0.44	-0.52 *	-0.52 *	-0.65 **	0.51 *
Tenacibaculum	-0.53 *	0.58 *	-0.54 *	0.19	-0.89 ***	-0.89 ***	-0.39	0.10
Balneola	0.13	0.33	0.16	-0.52 *	-0.31	-0.30	-0.65 **	0.59 *
Mamiellaceae	0.01	-0.02	-0.21	0.06	-0.26	-0.33	-0.43	0.30
Stramenopiles	-0.08	-0.15	0.01	0.16	0.34	0.47	0.14	-0.25
Synechococcus	0.69 **	-0.56 *	0.69 **	-0.33	0.44	0.29	-0.36	0.52 *
Rhodobacteraceae	-0.28	0.40	-0.58 *	0.07	-0.32	-0.45	0.20	-0.24
Ruegeria	-0.13	-0.37	0.01	0.53 *	0.43	0.52 *	0.53 *	-0.44
Other *Pelagibacteraceae*	0.39	0.12	-0.10	-0.70 **	0.03	-0.01	-0.09	0.07
Pelagibacter ubique	0.18	0.05	-0.30	-0.33	-0.10	-0.03	-0.08	-0.03
Altererythrobacter ishigakiensis	-0.32	-0.21	-0.05	0.72 **	0.12	0.37	0.27	-0.33

Table. S4 in Orita *et al*.

Table S4. Correlations between dominant sediment OTUs and environmental factors. For the dominant OTUs in the sediments, see Table 2. The correlation coefficient is calculated using Spearman's rank correlation coefficien (***: P>0.001, **: P>0.01, *: P>0.05).

OTU	Mud content	ORP	AVS	TOC	TN	C/N
OS-K	-0.40	0.80	0.21	-0.40	0.00	0.00
Other *Bacteroidetes*	1.00	-0.80	0.32	-0.40	0.20	-0.20
Bacteroidales	0.80	-0.40	-0.21	0.00	-0.40	0.40
GCA004	0.40	-0.80	-0.21	0.40	0.00	0.00
Stramenopiles	-1.00	0.80	-0.32	0.40	-0.20	0.20
Fusibacter	0.40	-0.20	-0.74	0.60	-0.80	0.80
Desulfococcus	-0.80	0.40	0.21	0.00	0.40	-0.40
Desulfobulbaceae	-0.80	1.00	-0.32	0.20	-0.40	0.40
Other *Deltaproteobacteria*	-0.80	0.40	0.21	0.00	0.40	-0.40
Desulfuromonadaceae	0.20	-0.40	-0.74	0.80	-0.60	0.60
Helicobacteraceae	0.20	-0.40	-0.74	0.80	-0.60	0.60
Sulfurimonas	0.20	-0.40	-0.74	0.80	-0.60	0.60
Other *Gammaproteobacteria*	-0.20	0.40	0.74	-0.80	0.60	-0.60
Chromatiales	-1.00	0.80	-0.32	0.40	-0.20	0.20
Piscirickettsiaceae	-0.40	0.80	0.21	-0.40	0.00	0.00
Photobacterium	0.32	-0.32	-0.78	0.74	-0.74	0.74
Marinicellaceae	-0.40	0.80	0.21	-0.40	0.00	0.00

Fig. S1 in Orita *et al*.

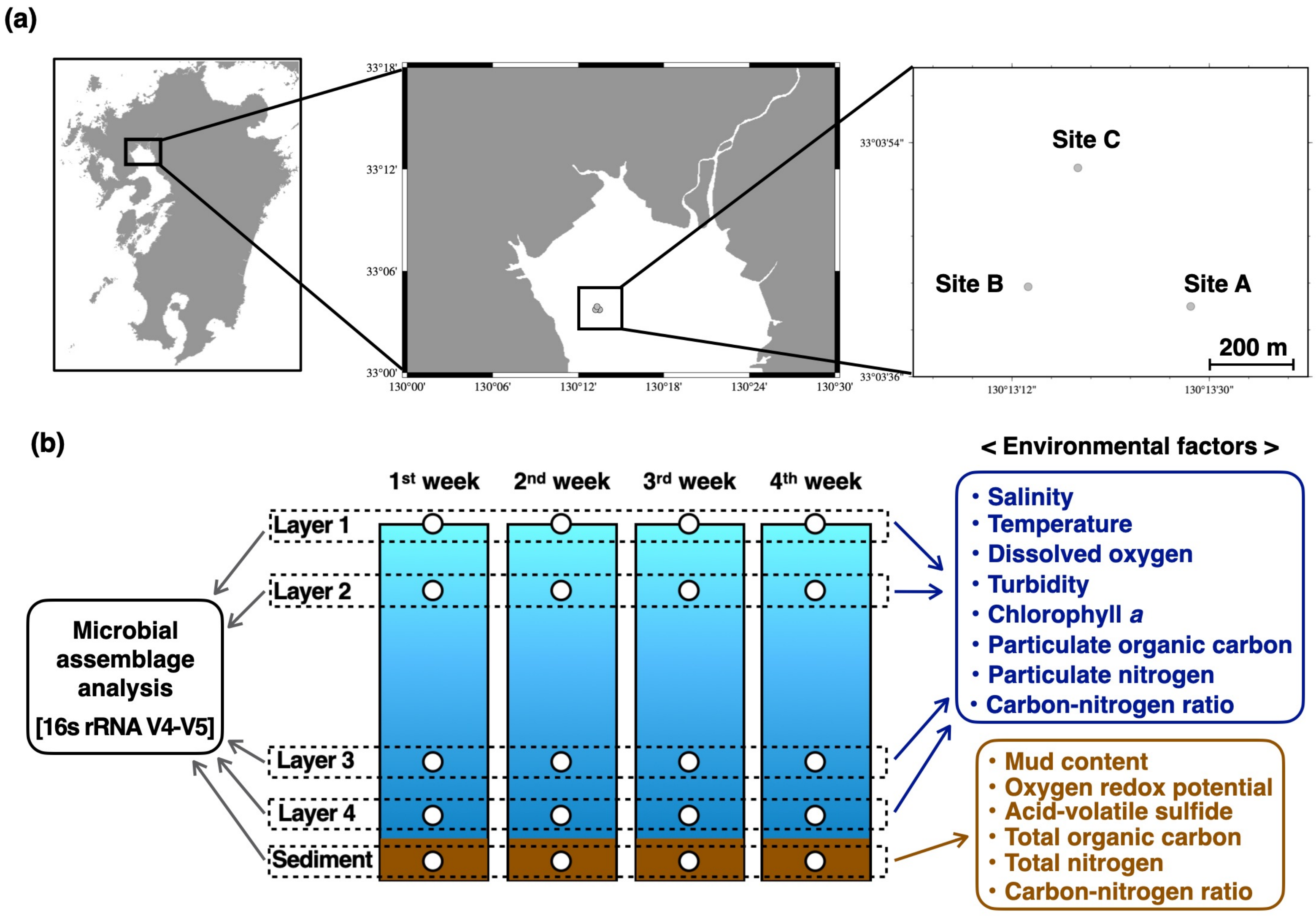

Fig. S1. Filed sampling scheme. (a) Sampling sites. The microbial assemblage analysis was based on samples from site B and the environmental factors were averaged over the three sites. (b) The layers examined to determine the relationship between the microbial assemblage and environmental factors (layer 1: sea surface, layer 2: 2 m in depth, layer 3: 2 m above the sea floor, layer 4: 0.5 m above the sea floor, and sediment surface: 0.5 cm in depth). White circles indicate the sampling points.

Fig. S2 in Orita *et al*.

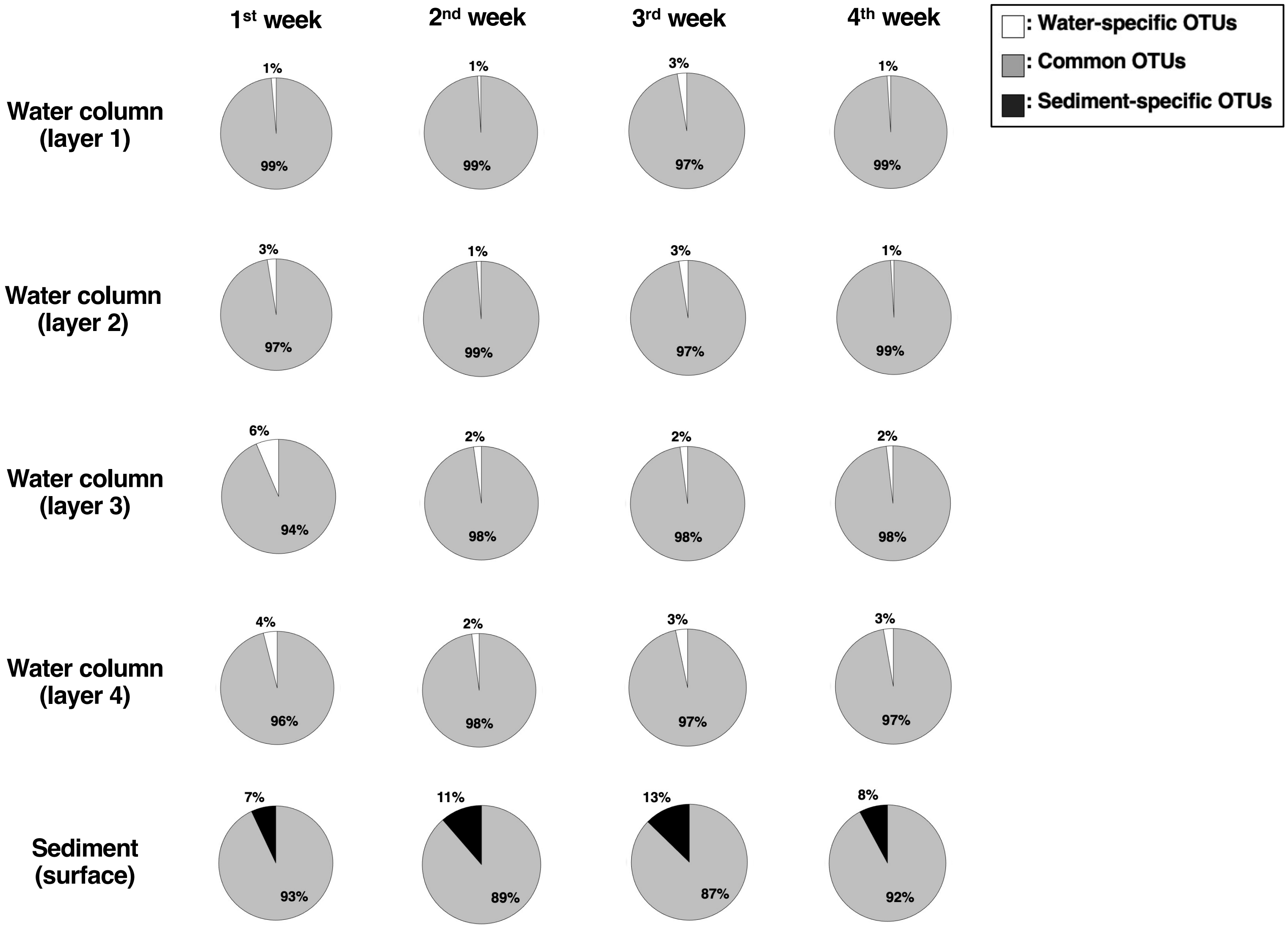

Fig. S2. Percentage of OTUs specifically occurring in the water column and the sediment relative to the microbial assemblages. For the explanation of sampling date and layer, see the legend to Figure 1.

Fig. S3 in Orita *et al*.

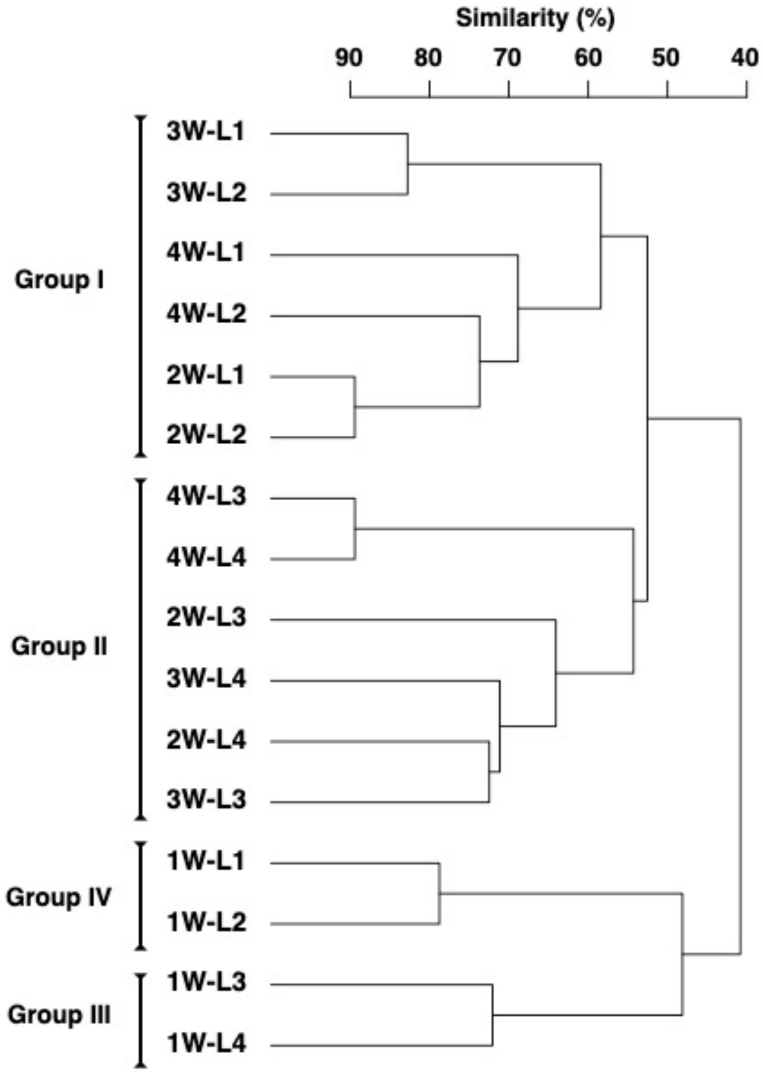

Fig. S3. Dendrogram of the microbial assemblages in the four layers of the water column during the monitoring period. In the label, the number before "W" indicates the week in which sampling was conducted, and the number after "L" indicates the layer that was sampled; "S" refers to sediment.

Fig. S4 in Orita *et al*.

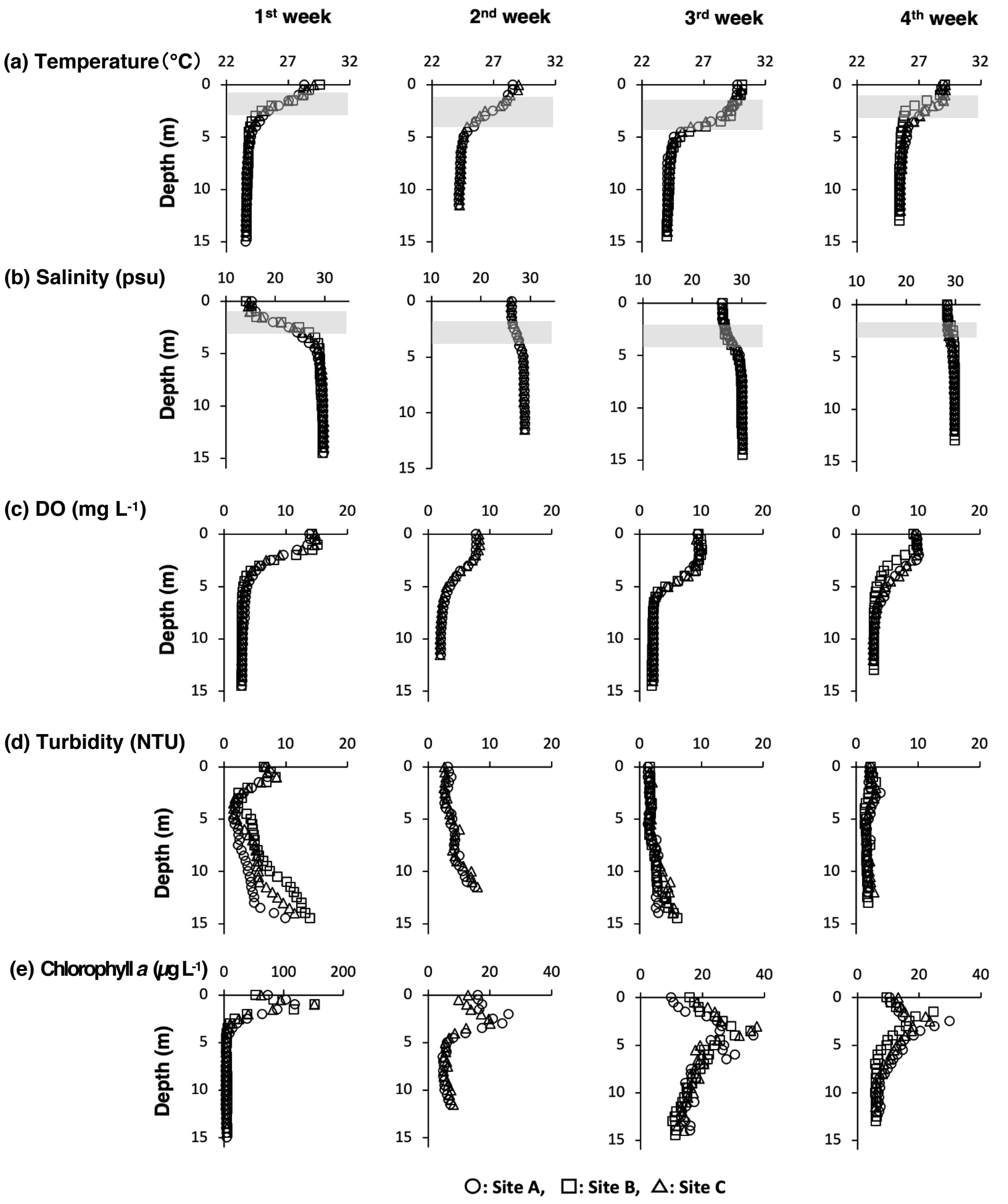

Fig. S4. Vertical profiles of environmental factors in the water column at the three sites. See Fig. S1 for information on sites. Grey shaded zones indicate the location of thermoclines and haloclines.
